# Clopidogrel-Induced Neutropenia after Coronary Stenting: Is Cilostazol a Good Alternative?

**DOI:** 10.1155/2011/867964

**Published:** 2011-08-11

**Authors:** Massimo Montalto, Italo Porto, Antonella Gallo, Claudia Camaioni, Roberta Della Bona, Antonio Grieco, Filippo Crea, Raffaele Landolfi

**Affiliations:** ^1^Institute of Internal Medicine, Catholic University of Rome, 00168 Rome, Italy; ^2^Institute of Cardiology, Catholic University of Rome, 00168 Rome, Italy; ^3^Department of Cardiovascular Medicine, Catholic University of Rome, Largo A. Gemelli 1, 00168 Rome, Italy

## Abstract

Dual antiplatelet therapy with aspirin plus thienopyridines has become the standard treatment of patients undergoing coronary stenting. Clopidogrel has mostly replaced the use of ticlopidine due to its more favourable adverse event profile. However, also the use of clopidogrel is not without side effects. Clopidogrel major adverse events are represented by marrow suppression, manifesting with aplastic anaemia, thrombocytopenia and neutropenia. When clopidogrel toxicity occurs, there are few and unsubstantiated alternative treatments and thus, in these cases, medical decisions may be very difficult. We report a case of clopidogrel-induced bone marrow toxicity manifesting with severe neutropenia in a patient treated with multiple coronary stents and provide suggestions for an alternative treatment.

## 1. Introduction

Dual antiplatelet therapy with aspirin plus clopidogrel has become the standard treatment of patients undergoing coronary stenting [1, 2]. Clopidogrel exerts its antiplatelet effect by inhibiting the binding of adenosine diphosphate (ADP) to its receptor (P2Y12) and consequent ADP-mediated platelet activation [3]. Current guidelines of the European Society of Cardiology suggest starting clopidogrel therapy before any percutaneous coronary intervention (PCI) or when acute coronary syndrome (ACS) occurs [4]. Clopidogrel use is associated with an increased risk of bleeding and may cause haematological adverse effects, such as thrombotic thrombocytopenic purpura, haemolytic uremic syndrome, and bone-marrow suppression, manifesting with aplastic anaemia, thrombocytopenia [5], and neutropenia [6, 7]. Minor side effects are represented by diarrhea, vomiting, hepatocellular injury, and skin rash [8–10]. It must be stressed that all these side effects occur a very low rate [4, 11]. The CAPRIE trial, which included 9.599 patients treated with clopidogrel, showed a low annual incidence (0.05%) of severe neutropenia, considered as neutrophil count below 0.45 × 10^9^/L [4]. Nevertheless, it is thought that the actual incidence of clopidogrel myelotoxicity could be somewhat underestimated [7].

When clopidogrel toxicity occurs, little is known about the efficacy and safety of alternative treatments, and thus, in these cases, medical decisions may be very difficult.

We report a case of clopidogrel-induced bone-marrow toxicity manifesting with severe neutropenia in a patient treated with multiple coronary stents and provide suggestions for an alternative treatment.

## 2. Case Report

A 65-year old Caucasian male was admitted to the Department of Internal Medicine of our Hospital for the sudden onset of palpable purpura on lower extremities. The patient was free from previous cardiovascular events, with untreated hypertension and smoker status as risk factors. He had no allergic history and denied any animal bite and recent ingestions of new drugs or diet components.

All haematological, microbiological, immunological, and allergic tests were normal. Skin biopsy revealed leukocytoclastic vasculitis; therefore, oral steroid therapy was initiated. The patient remained free from angina and dyspnoea; nevertheless, the ECG showed ischemic alterations in the lateral leads, and an echocardiogram showed hypokinesis of the septal, lateral, and anterior wall. Cardiac enzymes were within the normal range. Coronary angiogram, performed after administration of aspirin (100 mg) and of a 300 mg loading dose of clopidogrel, showed a critical stenosis of the left anterior descending artery involving the origin of the first diagonal branch and a severe short stenosis of a large, dominant circumflex artery at the level of the first marginal branch. Percutaneous coronary intervention (PCI) was then undertaken, which, due to the complex nature of the stenosis, required implantation of two drug-eluting stents (DESs) (Xience V, Abbot, Temecula, Calif, USA) on both bifurcations. A total of 45 mm of everolimus-eluting stent was used. 

After the procedure, clopidogrel (75 mg/day) and aspirin (100 mg/day) were prescribed as chronic treatment. Five weeks later, the patient came back to our Emergency Department complaining of fever and dyspnoea. Blood tests revealed severe leukopenia (white cells 1 × 10^3^/*μ*L) with marked neutropenia (neutrophils 3%), anaemia (haemoglobin 9.9 g/dL), and an important rise in inflammatory markers (C-reactive protein 122 mg/dL). A chest computed tomography revealed multiple inflammatory infiltrates. Cytological evaluation of bone-marrow aspirate showed hypoplasia of the granulocytic series with blocked maturation to the stage of promyelocytes. Clopidogrel-induced neutropenia was suspected, and therapy was immediately discontinued. Application of the Naranjo Causality Scale classified the adverse drug reaction as probable [11]. Empiric antibiotic therapy with levofloxacin (500 mg twice daily) was started and continued for one week. Granulocyte colony-stimulating factor (G-CSF) (5–10 *μ*/Kg/die) was administered for 72 hours. In the following ten days, total leucocytes and neutrophils count increased and returned within the normal range.

**Figure 1:**
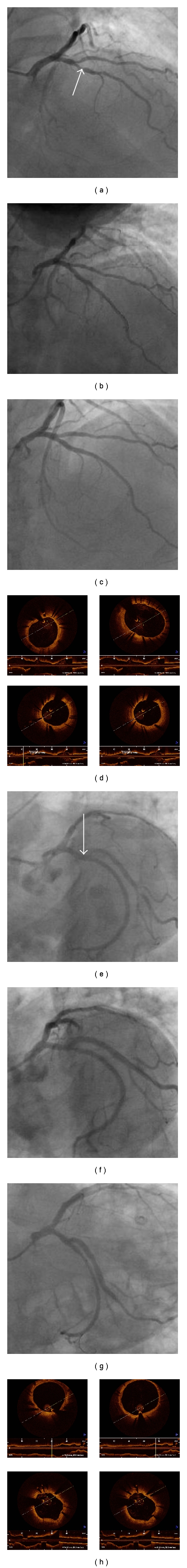
Upper panel shows the left coronary angiogram in the standard right anterior oblique cranial view. (a) At baseline, anterior descending artery (LAD) demonstrates a critical stenosis involving the origin of the first diagonal branch. (b) After two stents implantation in a “t and small protrusion”(TAP) fashion, good angiographic result can be seen. (c) One year angiographic followup attests that good result is maintained. (d) optical coherence tomography (OCT) analysis of LAD and diagonal branch (4 representative slices) at one year shows good neointimal coverage of the stent struts, without “malapposition”. Lower panel shows the left coronary angiogram in the standard left anterior oblique view. (e) At baseline, critical stenosis of the circumflex artery (LCX) at the level of the first marginal branch is visible. (f) After two stents implantation in a “culotte” fashion, good angiographic result can be seen. (g) One year angiographic followup testifies the maintenance of good angiographic result. (h) optical coherence tomography (OCT) analysis of LAD and diagonal branch (4 representative slices) at one year shows good neointimal coverage of the stent struts, without “malapposition”.

In consideration of the perceived high risk of stent thrombosis, it was necessary to replace clopidogrel with another drug in order to ensure adequate platelet inhibition. Intravenous infusion of eptifibatide (2 *μ*g/kg/min) was added to aspirin [12]. Moreover, anticoagulation was started, initially with enoxaparin (100 UI/Kg twice daily) and then with oral anticoagulants (dicumarol, titrated to obtain an International Normalised Ratio of 2-3). A week later, eptifibatide infusion was discontinued, and cilostazol 100 mg twice daily was started. Cilostazol is an antiplatelet that selectively inhibits cyclic nucleotide phosphodiesterase type 3 (PDE III) [13] and is commonly used for the management of the peripheral arterial disease. In the following days, the clinical conditions of the patient progressively improved. Neutrophil count remained within normal range, and the patient was discharged after six days. One year after the start of the combination therapy with aspirin, cilostazol, and oral anticoagulants, our patient remained asymptomatic. Repeat angiogram was performed, which showed absence of restenosis or plaque progression. Additionally, optical coherence tomography (OCT) showed complete neointimal coverage of the four implanted stents [14] (Figure 1). Cilostazol was thus stopped. Three months after cilostazol withdrawal, the patient is still in good health.

## 3. Discussion

Severe neutropenia has been reported as a rare adverse effect of thienopyridines, usually occurring from four weeks to three months after the start of therapy [3]. Neutropenia is less frequent with clopidogrel than with ticlopidine, as the latter directly inhibits colony-forming unit (CFU-C) replication in the bone marrow in a dose-dependent manner [15]. In our patient, clopidogrel was considered the most likely causative agent responsible for this severe haematological injury, as after few days from its withdrawal and after only three days of G-CSF therapy, neutrophil count rose and remained within the normal range for the following months [11].

Having to choose a new antiplatelet drug, we excluded the potential usage of another P2Y12 inhibitor due to the inherent risk of hematologic toxicity linked to drugs belonging to the same family. Ticlopidine was felt to be unsuitable for our patient due the well-known risk of bone-marrow complications reported for this drug [3, 16], and we also excluded the newly introduced P2Y12 inhibitor prasugrel for a possible cross-reaction. At the moment of the event, the nonthienopyridine ticagrelor had not been tested in large studies. Thus, we selected cilostazol. 

Our choice was influenced by the favourable pharmacodynamic characteristics of the drug. Cilostazol is a PDEIII isozyme selective inhibitor which potently inhibits the activities of PDEIIIA, the cardiovascular PDEIII subtype, thus increasing the cyclic AMP (cAMP) content of human platelets. Additionally, cilostazol has another important pharmacological property: the blockage of platelet adenosine uptake. Intracellular cAMP is degraded via several PDE and synthesized by adenylate cyclase (AC). AC activity in turn is controlled by stimulatory (Gs) and inhibitory (Gi) G-proteins. Adenosine, either from cellular metabolism or extracellular sources, activates Gs via A2-receptors and Gi via A1-receptors. Platelets and vascular cells that on their surface expose A2-receptors, thus, after inhibition of adenosine uptake, increase their cAMP cytoplasmatic levels. Therefore, cilostazol increases cAMP levels in platelets thanks to both PDE inhibition and blockage of adenosine uptake [13]. 

The most commonly reported side effects for cilostazol in clinical trials are those related to the vasodilator effect of the drug, namely, headache (primarily responsible for the suspension of the treatment) dizziness, and peripheral edema. Gastrointestinal symptoms such as diarrhea are also common. Cardiovascular adverse events consist of palpitations, arrhythmias, and ventricular extrasystoles [17]. One hundred mg twice daily is the recommended dose.

In 2007, Schäfer et al. reported the occurrence of severe neutropenia under clopidogrel treatment three weeks after coronary stenting in a 65-year-old woman [18]. Since this patient was considered at very high risk of stent thrombosis, association treatment with aspirin (150 mg/d) and dipyridamole (200 mg/bid) was started. Also, oral anticoagulant therapy was added, because impaired left ventricular function was present [19]. Available followup, however, was only two months.

In our case, similar to Schäfer's team, we opted for anticoagulation [18, 19], since left ventricular function was reduced; nevertheless, we introduced cilostazol instead of dipyridamole. 

Our preference of cilostazol was conditioned by two factors: (1) cilostazol inhibits platelets more effectively because its action involves with two mechanisms (PDE inhibition and blockage of adenosine uptake); (2) on clinical grounds, dipyridamole has never been tested as a part of antiplatelet therapy after PCI with DES, whereas cilostazol has been used in several studies. 

A large meta-analysis published in 2008 reviewed 23 randomized clinical trials (RCTs) for a total of 5428 patients. Coprimary end points were angiographic restenosis and repeat revascularization. Results showed the effectiveness of cilostazol in reducing restenosis rate and repeat revascularization after PCI with DES; furthermore, cilostazol also appeared to be safe, with no significant increase in the risk of stent thrombosis or bleeding [20]. 

Cilostazol has been tested as addition to the usual dual antiplatelet therapy (DA) to reduce the risk of stent thrombosis. A Korean study (2009) investigated the effectiveness of the triple antiplatelet therapy (TA) (aspirin, clopidogrel, plus cilostazol) in diabetic patients undergoing coronary stent. 55 type II diabetic patients DES-treated were stratified to receive DA (*n* = 34) and TA (*n* = 21). Platelet aggregation was studied with adenosine diphosphate (ADP) stimulation, and then, the antiplatelet power was compared using light transmittance aggregometry between groups. Results showed that TA was more potent than the DA. These findings suggest that TA may be more effective in preventing thrombotic complications after DES implantation in type 2 diabetic patients [21]. Similar conclusions, regardless of diabetes, arise from Tamhane's meta-analysis that analyzed 10 RCTs (*n* = 2,809 patients) comparing TA with standard DA: antiplatelet therapy with cilostazol was associated with a significant reduction in angiographic restenosis [22]. Another meta-analysis, including 1457 post-PCI patients, studied not only the angiographic restenosis rate, but also the frequency of major adverse cardiac and/or cerebrovascular events (MACE/MACCE), stent thrombosis and bleeding in TA versus DA, after a median follow-up period of 6–9 months. Coprimary end points were not only angiographic restenosis, but also the rates of major adverse cardiac and/or cerebrovascular events (MACE/MACCE), stent thrombosis, and bleeding in TA versus DA. This careful analysis showed that cilostazol was effective in reducing angiographic restenosis without any significant benefit for MACE/MACCE rates [23]. The CILTON-T trial (960 DES-treated patients enrolled and randomized to receive DA or TA) shows more disappointing results: despite the greater reduction of platelet reactivity by addition of cilostazol to conventional DA therapy, TA did not show superiority in reducing cardiac death, nonfatal myocardial infarction, ischemic stroke, and target lesion revascularization [24]. However, TA appeared to have a beneficial clinical effect in an higher-risk population (patients with acute ST-segment elevation myocardial infarction (STEMI) undergoing PCI). A total of 4203 STEMI treated by DES were analyzed retrospectively. They received either DA (*n* = 2569) or TA (*n* = 1634). MACE after 8 months were significantly lower in the group with TA [25]. 

Of note, other studies tested the effectiveness and the safety of cilostazol as a substitute for clopidogrel in association with aspirin (the drug combination we used in our patient). A study on 280 patients showed that combination therapy with aspirin and cilostazol for the prevention of stent thrombosis (after only 1 month of aspirin, cilostazol, and clopidogrel combination treatment) was comparable or superior to aspirin and clopidogrel in diabetic patients undergoing DES implantation [26]. In particular, the risk of late stent thrombosis, in this complex population with high baseline risk, was not increased. In a larger population (689 patients), another team also obtained the same results although the patients were all treated with BMS [27]. More recently, a large, randomised Korean study in 1315 DES-treated patients proved the efficacy of cilostazol in the prevention of stent thrombosis. During the initial six months after PCI, patients were randomized to TA or DA (aspirin + clopidogrel); then, after this first period, all patients were randomized to DA with aspirin and cilostazol or DA with aspirin and clopidogrel [28]. Results were encouraging for cilostazol both in TA (association) and in DA (alternative) for the prevention of stent thrombosis. 

As our strategy led to a successful long-term outcome for a difficult patient, we speculate that cilostazol might constitute an acceptable alternative treatment when thienopyridines are absolutely contraindicated, such as in cases of bone marrow toxicity. Further large-scale, randomized trials, however, are needed to fully elucidate this topic.
